# How does empagliflozin improve arterial stiffness in patients with type 2 diabetes mellitus? Sub analysis of a clinical trial

**DOI:** 10.1186/s12933-019-0839-8

**Published:** 2019-03-29

**Authors:** Agnes Bosch, Christian Ott, Susanne Jung, Kristina Striepe, Marina V. Karg, Dennis Kannenkeril, Thomas Dienemann, Roland E. Schmieder

**Affiliations:** 1 Department of Nephrology and Hypertension, Friedrich-Alexander-University Erlangen-Nürnberg (FAU), Erlangen, Germany; 2 Paracelsus Medical School Nürnberg, Nuremberg, Germany; 3 Department of Cardiology, Friedrich-Alexander-University Erlangen-Nürnberg (FAU), Erlangen, Germany

**Keywords:** Diabetes mellitus type 2, Empagliflozin, Vascular function, Central hemodynamics, Inflammation

## Abstract

**Background:**

Empagliflozin has been shown to reduce cardiovascular mortality, but the underlying pathogenetic mechanisms are poorly understood. It was previously demonstrated that empagliflozin improved arterial stiffness.

**Methods:**

Our analysis comprising 58 patients with type 2 diabetes mellitus identifies factors triggering the improvement of arterial stiffness. All patients participated in an investigator-initiated, prospective, double-blind, randomized, placebo-controlled, interventional clinical trial (http://www.ClinicalTrials.gov: NCT02471963, registered 15th June 2015, retrospectively registered) and received either 6-weeks treatment with 25 mg empagliflozin orally once daily or placebo (crossover). Central systolic pressure and central pulse pressure were recorded by the SphygmoCor System (AtCor Medical). Now, we investigated the impact of parameters of glucose metabolism, volume status, sympathetic activation, lipids, uric acid, blood pressure and inflammation on vascular parameters of arterial stiffness using multivariate regression analysis.

**Results:**

As previously reported, therapy with empagliflozin improved arterial stiffness as indicated by reduced central systolic blood pressure (113.6 ± 12.1 vs 118.6 ± 12.9 mmHg, p < 0.001), central pulse pressure (39.1 ± 10.2 vs 41.9 ± 10.7 mmHg, p = 0.027) forward (27.1 ± 5.69 vs 28.7 ± 6.23 mmHg, p = 0.031) as well as reflected wave amplitude (18.9 ± 5.98 vs 20.3 ± 5.97 mmHg, p = 0.045) compared to placebo. The multivariate regression analysis included age, sex and change between empagliflozin and placebo therapy of the following parameters: HbA1c, copeptin, hematocrit, heart rate, LDL-cholesterol, uric acid, systolic 24-h ambulatory blood pressure and high sensitive CRP (hsCRP). Besides the influence of age (beta = − 0.259, p = 0.054), sex (beta = 0.292, p = 0.040) and change in systolic 24-h ambulatory blood pressure (beta = 0.364, p = 0.019), the change of hsCRP (beta = 0.305, p = 0.033) emerged as a significant determinant of the empagliflozin induced reduction in arterial stiffness (placebo corrected). When replacing HbA1c with fasting plasma glucose in the multivariate regression analysis, a similar effect of the change in hsCRP (beta = 0.347, p = 0.017) on arterial stiffness parameters was found.

**Conclusion:**

Besides age and sex, change in systolic 24-h ambulatory blood pressure and change in hsCRP were determinants of the empagliflozin induced improvement of vascular parameters of arterial stiffness, whereas parameters of change in glucose metabolism and volume status had no significant influence. Our analysis suggests that empagliflozin exerts, at least to some extent, its beneficial vascular effects via anti-inflammatory mechanisms.

*Trial registration* http://www.ClinicalTrials.gov: NCT02471963, registered 15th June 2015, retrospectively registered

## Introduction

Treatment of type 2 diabetes should aim at improving vascular structure and function in the micro- and macrocirculation besides metabolic control [1]. Arterial stiffness, a key parameter of vascular changes, is characterized by an increased pulse wave velocity along the arterial tree of both the forward and backward (reflected) pulse wave leading to increased central systolic blood pressure and elevated central pulse pressure [2, 3]. Central systolic blood pressure is the integral of various components of arterial stiffness, an important surrogate parameter of afterload, and strongly linked to future cardiovascular outcome [4, 5]. Likewise, central pulse pressure has been shown to be superior in the prediction of cardiovascular events compared to measurements of pulse pressure at the brachial level, and there is evidence for an association between both forward and backward wave amplitudes and increased risk for incident cardiovascular disease and all-cause mortality [5–7].

In the EMPA-REG OUTCOME study (Empagliflozin Cardiovascular Outcome Event Trial in Type 2 Diabetes Mellitus Patients) treatment with the selective sodium-glucose cotransporter 2 inhibitor (SGLT2-inhibitor) empagliflozin reduced the primary combined cardiovascular end point as well as secondary end points of hospitalization due to heart failure, cardiovascular morbidity, total mortality and renal end points [8]. The underlying pathophysiologic mechanisms are currently under intensive discussion, but the crucial question about the pivotal mechanism causing the reduced cardiovascular death rate and total mortality still remains to be elucidated. Interestingly, the benefits observed in the EMPA-REG OUTCOME study were documented in a population in whom cardiovascular risk factors, including blood pressure and dyslipidemia were well treated with the use of renin–angiotensin–aldosterone system inhibitors, statins and acetylsalicylic acid. The authors of the EMPA-REG OUTCOME study mention changes in arterial stiffness among others as possible mechanisms [8]. Most recently we have shown that empagliflozin improves arterial stiffness in a double blind, placebo controlled, crossover clinical trial including 71 patients with type 2 diabetes mellitus [9]. The aim of the current analysis is to identify potential determinants for the improvement of arterial stiffness observed during empagliflozin therapy.

## Methods

### Study design

This is a prespecified analysis of patients, who participated in an investigator initiated prospective, double blind, randomized, placebo-controlled, cross-over, interventional single center study conducted at the Clinical Research Center of the Department of Nephrology and Hypertension, University of Erlangen-Nuremberg, Germany (http://www.crc-erlangen.de) (http://www.ClinicalTrials.gov: NCT02471963). The principal findings of the clinical trial have been previously published [9]. Participants were recruited by advertising in local newspapers in the area of Erlangen-Nuremberg, Germany, and eligible participants were enrolled consecutively. Written informed consent was obtained before study inclusion. The study protocol was approved by the local ethics committee (University of Erlangen-Nuremberg), and the study was conducted in accordance with the Declaration of Helsinki and the principles of good clinical practice guidelines.

### Analysis of changes in variables

On the basis of evidence from previous studies [10] the following mediators involving several mechanistic categories have been chosen for analysis: Glucose control (HbA1c, fasting plasma glucose), volume status (copeptin, hematocrit), sympathetic activation (heart rate), lipids (LDL-cholesterol), vascular tone (systolic 24-h ambulatory blood pressure), inflammation [high sensitive CRP (hsCRP)] and other (uric acid).

### Study population

Characteristics of the study population have been previously published [9]. In brief, female and male patients aged between 18 and 75 years with diagnosed type 2 diabetes mellitus, defined by fasting glucose ≥ 126 mg/dl or HbA1c ≥ 6.5% (48 mmol/mol) or on blood glucose lowering medication, were included in the study. Estimated glomerular filtration rate (eGFR) had to be ≥ 60 ml/min/1.73 m^2^. Patients who used insulin, glitazone, gliptine or SGLT-2 inhibitor therapy within the past 3 months and patients with more than one oral blood glucose lowering medication were excluded. Patients on any antidiabetic agent had at least a 4 weeks wash-out phase prior to the baseline examination. Other key exclusion criteria were HbA1c ≥ 10% (86 mmol/mol), fasting plasma glucose > 240 mg/dl, any history of stroke, transient ischemic attack, instable angina pectoris or myocardial infarction within the last 6 months prior to study inclusion, uncontrolled hypertension (office blood pressure ≥ 180/110 mmHg), congestive heart failure (CHF) NYHA stage III and IV, use of loop diuretics and pregnancy. Eight patients from the original study cohort (71 patients) were excluded because they presented with hsCRP values above 5 mg/l. Another five patients from the original study cohort showed a clinical infect correlate such as cystitis, vaginal infection, cold or gout. Even though these patients did not present with hsCRP above 5 mg/dl, they were excluded from our analysis based on the clinical investigation. Conventional blood pressure and heart rate measurements in the office and during 24-h were carried in standard fashion by validated devices.

### Treatment

Patients underwent a run-in/wash-out phase of 4 weeks if pretreated with any antidiabetic agent, or 2 weeks if not pretreated with any antidiabetic agent and afterwards were randomized to either empagliflozin 25 mg orally once daily or placebo. Following 6 weeks of treatment with either of these drugs, the patient underwent a wash-out phase of 1 week. Then the patient received the other substance for another 6 weeks of intervention (cross-over).

### Assessment of vascular function and central hemodynamics

To derive the central (aortic) arterial waveform, a validated system (SphygmoCorTM System; AtCor Medical, Sydney, Australia) was applied [5, 7, 20] by recording radial artery waveforms from the radial artery at the wrist, using high-fidelity applanation tonometer (Millar Instruments, Houston, Tex.) [5, 6, 20]. Corresponding central (aortic) waveforms were then automatically generated from the radial artery waveform by a validated transfer function [5, 7]. This allows obtainment of the following parameters: central systolic pressure, central pulse pressure, central augmentation pressure, central augmentation index (cAIx), cAIx normalized to a heart rate of 75 beats per minute (cAIx@75), pulse pressure amplification, as well as forward and backward reflected wave amplitude.

### Assessment of blood pressure and potential determinants of vascular function

Office blood pressure measurement was performed in a standardized fashion according to guideline recommendations [4]. During 24-h ambulatory daily-life conditions, brachial systolic and diastolic blood pressure, pulse pressure and heart rate were measured by the Mobilograph (IEM, Aachen, Germany). The technology has been validated previously [5, 11, 12].

All blood samples were measured centrally at the biochemistry laboratory of the University of Erlangen-Nuremberg according to established methods. In particular, hsCRP was measured via particle-reinforced nephelometry. Copeptin was analysed by lab MVZ Dr. Limbach GbR using Time Resolved Amplified Cryptate Emission method. Coefficient of variation of measurements was below 10%.

### Statistical methods

Normal distribution of data was confirmed by histogram and Kolmogorov–Smirnov test prior to further analysis. Data were compared by paired and unpaired t-tests and expressed as mean ± standard deviation (SD) in text and tables. A two-sided p-value < 0.05 was considered statistically significant. Bivariate correlation analyses were performed using Pearson’s test. Multivariate regression analysis was performed including the parameters sex, age and change of the following parameters under treatment with empagliflozin: HbA1c (model 1), copeptin concentration, hematocrit, 24-h ambulatory heart rate, LDL-cholesterol, uric acid, 24-h ambulatory blood pressure and hsCRP. A second multivariate regression analysis model included besides the other previously mentioned parameters fasting plasma glucose instead of HbA1c (model 2). Vascular stiffness parameters entered our model as an independent variable, namely as first change in central systolic blood pressure, second change in pulse pressure, third change in forward wave amplitude and fourth change in reflected wave amplitude. A separate multiple regression analysis was performed for each of the four independent variables mentioned. Potential collinearity between the dependent variables in our model has been excluded by calculating correlation coefficients between the dependent variables. There is no correlation between change in systolic 24-h ambulatory blood pressure and sex (r = − 0.006, p = 0.967), age (r = − 0.008, p = 0.955) and change of the following parameters: uric acid (r = 0.104, p = 0.443), hsCRP (r = − 0.027, p = 0.844), LDL-cholesterol (r = − 0.204, p = 0.129), fasting plasma glucose (r = 0.165, p = 0.220), hematocrit (r = 0.93, p = 0.490) and copeptin (r = − 0.074, p = 0.591). All analyses were performed using IBM SPSS Statistics 22 (SPSS Inc, Chicago, IL/USA).

## Results

### Study population

Characteristics of the study cohort have been previously published [9]. In brief, the study cohort comprised 58 patients with type 2 diabetes mellitus (all Caucasians, 59% male) with mean age of 62 ± 7 years, HbA1c level of 6.69 ± 0.8% (50 ± 8.7 mmol/mol), office blood pressure 128 ± 13/78 ± 7.2 mmHg, 24-h ambulatory blood pressure 129 ± 10/79 ± 6.3 mmHg, body weight 87.7 kg and body mass index of 29.5 ± 3.9 kg/m^2^. None of the patients were on any antidiabetic medication (85% were on metformin prior to study inclusion), whereas 50 patients received antihypertensive medications at baseline (84% received an angiotensin receptor blocker or an ACE-inhibitor), without any changes in medication throughout the study period.

### Influence of empagliflozin therapy

Consistent with our previous results [9], after therapy with empagliflozin there was a decrease in HbA1c (p < 0.001), fasting plasma glucose (p < 0.001), body weight (p < 0.001), brachial office blood pressure (p < 0.001/p = 0.002), 24-h ambulatory blood pressure (p = 0.021/p = 0.007), central systolic blood pressure (p < 0.001) and central pulse pressure (p = 0.027) forward (p = 0.031) and backward (reflected) wave amplitude (p = 0.045) compared to placebo (Table 1).

**Table 1:** Effect of empagliflozin on metabolic parameters

Parameter	Baseline	EMPA	Placebo	EMPA vs baseline	Placebo vs baseline	Verum vs placebo
HbA1c (%)	6.69 ± 0.82	6.64 ± 0.76	6.89 ± 1.03	< 0.001	< 0.001	< 0.001
HbA1c (mmol/mol)	50 ± 9.0	49 ± 8.3	52 ± 11.3	< 0.001	< 0.001	< 0.001
Fasting plasma glucose (mg/dl)	136.5 ± 31.4	115.1 ± 19.7	139.2 ± 40.7	< 0.001	0.384	< 0.001
Body weight (kg)	87.7 ± 12.9	86.6 ± 12.6	87.6 ± 12.9	< 0.001	0.858	< 0.001
Systolic OBP (mmHg)	128 ± 13.4	122 ± 11.4	128 ± 12.7	< 0.001	0.878	< 0.001
Diastolic OBP (mmHg)	77.7 ± 7.2	75 ± 6.7	78 ± 7.9	< 0.001	0.999	0.002
Systolic 24-h ABP (mmHg)	129 ± 10.4	127 ± 9.8	129 ± 9.7	0.053	0.868	0.021
Diastolic 24-h ABP (mmHg)	79.2 ± 6.3	77.7 ± 6.9	80 ± 7.0	0.041	0.624	0.007
Central systolic BP (mmHg)	120.3 ± 12.8	113.6 ± 12.1	118.6 ± 12.9	< 0.001	0.237	< 0.001
Central PP (mmHg)	43.3 ± 11.5	39.1 ± 10.2	41.9 ± 10.7	< 0.001	0.248	0.027
FWA (mmHg)	28.9 ± 6.24	27.0 ± 5.64	28.7 ± 6.23	0.002	0.553	0.031
BWA (mmHg)	21.2 ± 6.48	18.9 ± 5.98	20.4 ± 6.01	< 0.001	0.248	0.045
Copeptin (pmol/l)	5.65 ± 4.12	6.81 ± 4.13	5.06 ± 2.83	0.001	0.211	< 0.001
Hematocrit (%)	41.5 ± 2.78	43.0 ± 2.75	42.2 ± 2.73	< 0.001	0.032	0.004
HR (bpm)	72.5 ± 9.17	71.8 ± 9.18	72.2 ± 10.3	0.439	0.787	0.577
Cholesterol (mg/dl)	178.1 ± 35.3	182.5 ± 39.0	180.3 ± 36.9	0.077	0.325	0.413
LDL-cholesterol (mg/dl)	88.0 ± 24.2	92.9 ± 27.4	91.2 ± 24.3	0.016	0.061	0.425
HDL-cholesterol (mg/dl)	52.2 ± 11.3	54.5 ± 12.9	53.4 ± 11.8	< 0.002	0.086	0.219
Uric acid (mg/dl)	6.05 ± 1.26	4.85 ± 1.27	5.89 ± 1.23	< 0.001	0.061	< 0.001
eGFR (ml/min/1.73 m^2^)	92.7 ± 6.98	91.2 ± 7.42	92.5 ± 7.01	< 0.001	0.535	< 0.001
hsCRP (mg/l)	2.10 ± 1.72	1.99 ± 1.19	1.88 ± 1.32	0.583	0.283	0.458

Data are given as mean ± SD

*OBP* office blood pressure, *24-h ABP* 24 h ambulatory blood pressure, *PP* pulse pressure, *FWA* forward wave amplitude, *BWA* backward wave amplitude, *HR* heart rate, *LDL* low density lipid, *HDL* high density lipid, *eGFR* estimated glomerular filtration rate (calculated from serum creatinine using CKD-EPI formula), *hsCRP* high sensitive C-reactive protein

Further analysis now included volume parameters such as copeptin and hematocrit, parameters of glucose metabolism such as HbA1c and fasting plasma glucose, hsCRP as parameter of inflammation, LDL-cholesterol, uric acid, and heart rate as parameter of sympathetic activation (Table 1). Copepetin levels (p < 0.001) and hematocrit (p = 0.004) were higher in patients treated with empagliflozin compared to placebo (Table 1). Uric acid (p < 0.001) was lower in patients treated with empagliflozin compared to placebo (Table 1). No difference between empagliflozin and placebo therapy was observed in heart rate (p = 0.513), total cholesterol (p = 0.413) as well as HDL- (p = 0.219) and LDL-cholesterol (p = 0.425) and hsCRP (p = 0.458). Estimated glomerular filtration rate (p < 0.001) was significantly lower after treatment with empagliflozin compared to placebo (Table 1).

### Multivariate regression analysis

Model 1 of the multiple regression analysis identified change in systolic 24-h ambulatory blood pressure as the only significant determinant of change in central systolic blood pressure after therapy with empagliflozin (Tables 2 and 3). Besides change in hematocrit, change in 24-h ambulatory blood pressure was also a determinant of change in forward wave amplitude. Interestingly, change in hsCRP and change in systolic 24-h ambulatory blood pressure emerged besides age as significant determinants of change in central pulse pressure (Table 2). Besides the influence of change in systolic 24-h ambulatory blood pressure, there was a trend towards a significant influence of change in hsCRP on change in reflected wave amplitude (Table 3).

**Table 2:** Results of multivariate regression analysis

Dependent variable: Δ central systolic blood pressure						Dependent variable: Δ central pulse pressure					
Model 1	Beta	p-value	Model 2	Beta	p-value	Model 1	Beta	p-value	Model 2	Beta	p-value
Sex	0.134	0.379	Sex	0.195	0.191	Sex	0.155	0.255	Sex	0.211	0.110
Age	− 0.159	0.286	Age	− 0.186	0.205	Age	− 0.259	*0.054*	Age	− 0.282	*0.032*
Δ HbA1c	− 0.022	0.892	Δ Fasting plasma glucose	− 0.043	0.788	Δ HbA1c	0.012	0.933	Δ Fasting plasma glucose	− 0.169	0.238
Δ Copeptin	0.057	0.701	Δ Copeptin	0.054	0.710	Δ Copeptin	0.031	0.813	Δ Copeptin	0.037	0.769
Δ Hematocrit	− 0.092	0.565	Δ Hematocrit	− 0.096	0.556	Δ Hematocrit	− 0.145	0.309	Δ Hematocrit	− 0.189	0.192
Δ Heart rate	0.001	0.997	Δ Heart rate	0.043	0.787	Δ Heart rate	− 0.123	0.392	Δ Heart rate	− 0.056	0.686
Δ LDL-cholesterol	0.095	0.615	Δ LDL-cholesterol	0.074	0.631	Δ LDL-cholesterol	− 0.126	0.365	Δ LDL-cholesterol	− 0.146	0.287
Δ Uric acid	− 0.083	0.642	Δ Uric acid	− 0.027	0.878	Δ Uric acid	− 0.124	0.438	Δ Uric acid	− 0.112	0.478
Δ Systolic BP	0.412	*0.018*	Δ Systolic BP	0.355	*0.024*	Δ Systolic BP	0.364	*0.019*	Δ Systolic BP	0.332	*0.017*
Δ hsCRP	0.171	0.278	Δ hsCRP	0.192	0.231	Δ hsCRP	0.305	*0.033*	Δ hsCRP	0.347	*0.017*

Model 1: Multivariate regression analysis was performed including the parameters sex, age and change of the following parameters under treatment with empagliflozin: HbA1c (model 1), copeptin concentration, hematocrit, 24-h ambulatory heart rate, LDL-cholesterol, uric acid, systolic 24-h ambulatory blood pressure and hsCRP. Model 2 included besides the other previously mentioned parameters fasting plasma glucose instead of HbA1c. *LDL-cholesterol* low density lipid cholesterol, *hsCRP* high sensitive C-reactive protein, *heart rate* 24-h ambulatory heart rate, *systolic BP* systolic 24-h ambulatory blood pressure, “Δ” refers to the changes due to empagliflozin treatment corrected for the placebo changes

Italic values indicate significance of p-value (p < 0.05)

**Table 3:** Results of multivariate regression analysis

Dependent variable: Δ forward wave amplitude						Dependent variable: Δ reflected wave amplitude					
Model 1	Beta	p-value	Model 2	Beta	p-value	Model 1	Beta	p-value	Model 2	Beta	p-value
Sex	0.025	0.873	Sex	0.077	0.612	Sex	0.237	0.108	Sex	0.292	*0.040*
Age	− 0.180	0.243	Age	− 0.210	0.162	Age	− 0.194	0.169	Age	− 0.225	0.104
Δ HbA1c	0.009	0.960	Δ Fasting plasma glucose	− 0.155	0.366	Δ HbA1c	0.086	0.599	Δ Fasting plasma glucose	− 0.125	0.423
Δ Copeptin	0.095	0.528	Δ Copeptin	0.101	0.492	Δ Copeptin	0.008	0.955	Δ Copeptin	0.025	0.851
Δ Hematocrit	− 0.343	*0.054*	Δ Hematocrit	− 0.371	*0.037*	Δ Hematocrit	− 0.142	0.374	Δ Hematocrit	− 0.174	0.276
Δ Heart rate	− 0.134	0.435	Δ Heart rate	− 0.072	0.669	Δ Heart rate	− 0.159	0.311	Δ Heart rate	− 0.069	0.654
Δ LDL-cholesterol	0.083	0.615	Δ LDL-cholesterol	0.049	0.758	Δ LDL-cholesterol	− 0.095	0.531	Δ LDL-cholesterol	− 0.118	0.424
Δ Uric acid	0.047	0.809	Δ Uric acid	0.039	0.839	Δ Uric acid	0.046	0.794	Δ Uric acid	0.044	0.803
Δ Systolic BP	0.365	*0.049*	Δ Systolic BP	0.338	*0.038*	Δ Systolic BP	0.414	*0.016*	Δ Systolic BP	0.371	*0.014*
Δ hsCRP	0.188	0.257	Δ hsCRP	0.235	0.164	Δ hsCRP	0.279	0.070	Δ hsCRP	0.318	*0.043*

Model 1: Multivariate regression analysis was performed including the parameters sex, age and change of the following parameters under treatment with empagliflozin: HbA1c (model 1), copeptin concentration, hematocrit, 24-h ambulatory heart rate, LDL-cholesterol, uric acid, systolic 24-h ambulatory blood pressure and hsCRP. Model 2 included besides the other previously mentioned parameters fasting plasma glucose instead of HbA1c. *LDL-cholesterol* low density lipid cholesterol, *hsCRP* high sensitive C-reactive protein, *heart rate* 24-h ambulatory heart rate, *systolic BP* systolic 24-h ambulatory blood pressure, “Δ” refers to the changes due to empagliflozin treatment corrected for the placebo changes

Italic values indicate significance of p-value (p < 0.05)

In model 2 of the multivariate regression analysis change in systolic 24-h ambulatory blood pressure emerged as the only significant determinant of change in central systolic blood pressure, and besides hematocrit as the only determinant of change in forward wave amplitude after therapy with empagliflozin (Tables 2 and 3). Again, change in hsCRP and change in systolic 24-h ambulatory blood pressure emerged besides age as significant determinant of change in central pulse pressure and besides sex as significant determinant of change in backward (reflected) wave amplitude (Tables 2 and 3).

### Correlations

**Fig. 1:**
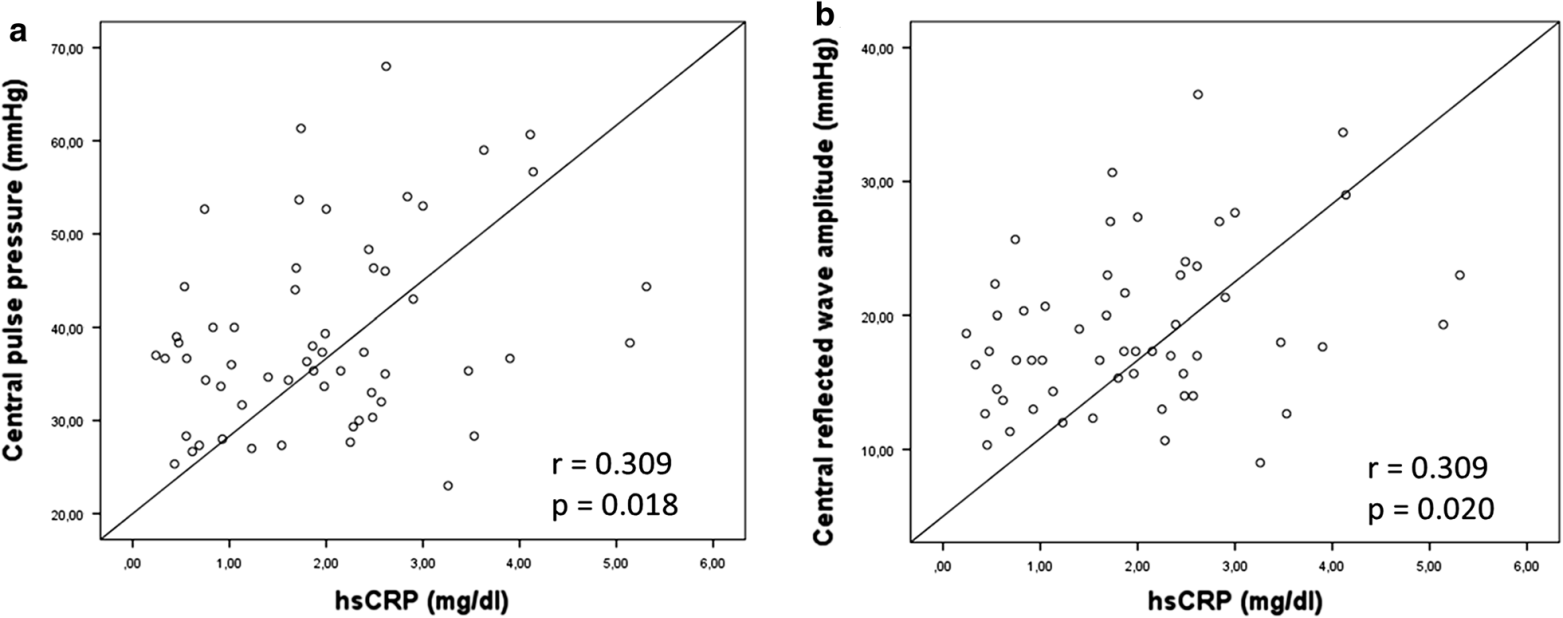
**a** Relation between hsCRP after 6 weeks treatment with empagliflozin and central pulse pressure after treatment with empagliflozin (r = 0.309, p = 0.018). **b** Relation between hsCRP after 6 weeks treatment with empagliflozin and reflected wave amplitude after treatment with empagliflozin (r = 0.309, p = 0.020)

There was a relation between central pulse pressure (r = 0.309, p = 0.018, Fig. 1a) as well as central reflected wave amplitude (r = 0.309, p = 0.020, Fig. 1b) and hsCRP 6 weeks after treatment with empagliflozin. No relation was found between central systolic blood pressure (r = 0.165, p = 0.217) as well as central forward wave amplitude (r = 0.183, p = 0.177) and hsCRP after empagliflozin treatment. No relation was present between placebo corrected changes in hsCRP and changes in central systolic blood pressure as well as changes in central pulse pressure, central forward and reflected wave amplitude (data not shown).

### Sub analysis of total study population

Dividing the study population according to median of age, baseline HbA1c, copeptin, 24-h ambulatory heart rate, LDL-cholesterol, uric acid, systolic 24-h ambulatory blood pressure and hsCRP at baseline did also not reveal any difference in change in central systolic blood pressure (Table 4), change in central pulse pressure (Table 5), change in forward (Table 6) and change in reflected wave amplitude (Table 7) after treatment with empagliflozin between the groups. However, when separating the study population according to median of baseline copeptin, patients above median showed a greater change in central systolic blood pressure (Table 4) and central pulse pressure (Table 5), suggesting that greater intravascular volume contraction was positively influencing some of the stiffness parameters.

**Table 4:** Mean change in central systolic blood pressure (mmHg) after EMPA therapy separated according to median of different variables

Variable	Age	HbA1c	Copeptin	Heart rate	LDL-cholesterol	Uric acid	Systolic BP	hsCRP
≥ median	− 6.23 ± 11.2	− 6.46 ± 11.1	− 8.94 ± 10.2	− 4.49 ± 9.89	− 5.50 ± 12.1	− 6.21 ± 9.54	− 5.13 ± 10.6	− 4.21 ± 10.3
< median	− 3.76 ± 9.83	− 3.61 ± 9.97	− 1.78 ± 9.83	− 5.48 ± 11.3	− 4.51 ± 9.06	− 3.76 ± 11.45	− 4.88 ± 10.7	− 5.77 ± 10.9
p-value	0.378	0.308	0.009	0.724	0.724	0.378	0.928	0.576

Data are given as mean ± SD

*hsCRP* high sensitive C-reactive protein, *LDL-cholesterol* low density lipid cholesterol, *heart rate* 24 h ambulatory heart rate, *systolic BP* systolic 24-h ambulatory blood pressure

**Table 5:** Mean change in central pulse pressure (mmHg) after EMPA therapy separated according to median of different variables

Variable	Age	HbA1c	Copeptin	Heart rate	LDL-cholesterol	Uric acid	Systolic BP	hsCRP
≥ median	− 4.66 ± 8.87	− 4.77 ± 9.04	− 5.54 ± 8.25	− 1.03 ± 9.37	− 1.95 ± 11.9	− 3.978 ± 9.39	− 2.65 ± 9.46	− 1.82 ± 8.11
< median	− 0.83 ± 9.27	− 0.84 ± 9.08	− 0.47 ± 9.43	− 4.45 ± 8.86	− 3.48 ± 5.82	− 1.51 ± 8.99	− 2.81 ± 9.13	− 3.67 ± 10.2
p-value	0.114	0.105	0.033	0.160	0.533	0.311	0.949	0.449

Data are given as mean ± SD

*hsCRP* high sensitive C-reactive protein, *LDL-cholesterol* low density lipid cholesterol, *heart rate* 24-h ambulatory heart rate, *systolic BP* systolic 24-h ambulatory blood pressure

**Table 6:** Mean change in forward wave amplitude (mmHg) after EMPA therapy separated according to median of different variables at baseline

Variable	Age	HbA1c	Copeptin	Heart rate	LDL-cholesterol	Uric acid	Systolic BP	hsCRP
≥ median	− 1.98 ± 5.31	− 2.14 ± 5.46	− 2.56 ± 5.60	− 1.86 ± 4.94	− 1.95 ± 6.47	− 2.23 ± 5.33	− 0.76 ± 5.10	− 1.31 ± 4.99
< median	− 1.19 ± 5.26	− 1.03 ± 5.07	− 0.81 ± 4.90	− 1.29 ± 5.65	− 1.27 ± 4.01	− 1.03 ± 5.21	− 2.30 ± 5.36	− 1.86 ± 5.58
p-value	0.585	0.442	0.225	0.692	0.638	0.406	0.287	0.708

Data are given as mean ± SD

*hsCRP* high sensitive C-reactive protein, *LDL-cholesterol* low density lipid cholesterol, *heart rate* 24-h ambulatory heart rate, *systolic BP* systolic 24-h ambulatory blood pressure

**Table 7:** Mean change in reflected wave amplitude (mmHg) after EMPA therapy separated according to median of different variables

Variable	Age	HbA1c	Copeptin	Heart rate	LDL-cholesterol	Uric acid	Systolic BP	hsCRP
≥ median	− 1.82 ± 4.93	− 2.25 ± 5.05	− 2.56 ± 4.54	− 0.88 ± 4.74	− 0.43 ± 5.86	− 1.94 ± 4.45	− 1.44 ± 5.18	− 0.26 ± 4.54
< median	− 0.83 ± 4.64	− 0.41 ± 4.36	− 0.35 ± 4.78	− 1.82 ± 4.84	− 2.11 ± 3.49	− 0.79 ± 5.03	− 1.24 ± 4.46	− 2.39 ± 4.83
p-value	0.449	0.159	0.091	0.471	0.199	0.382	0.877	0.101

Data are given as mean ± SD

*hsCRP* high sensitive C-reactive protein, *LDL-cholesterol* low density lipid cholesterol, *heart rate* 24-h ambulatory heart rate, *systolic BP* systolic 24-h ambulatory blood pressure

## Discussion

The SGLT-2 inhibitor empagliflozin recently emerged as a novel cardioprotective and nephroprotective treatment strategy [8, 13–15]. Empagliflozin reduced central systolic blood pressure and central pulse pressure, both important surrogate parameters strongly linked to future cardiovascular outcome which may serve to explain the reduction in cardiovascular mortality observed in the EMPA-REG OUTCOME study to some extent. Consistently, empagliflozin induced reduction of arterial stiffness has been previously reported in a post hoc analysis of data from five clinical trials [16]. We showed now that besides age and sex, change in systolic 24-h ambulatory blood pressure and change in hs CRP were determinants of the empagliflozin induced improvement of arterial stiffness parameters, whereas change in glucose metabolism and volume status were not related to the improvement of arterial stiffness following empagliflozin therapy.

Central systolic blood pressure is primarily determined by arterial stiffness of large arteries and was found to be independently associated with cardiovascular morbidity and mortality [3, 4, 17]. Increasing arterial stiffness leads to increased pulse wave propagation along the artery tree resulting in elevated carotid femoral pulse wave velocity and finally augmented central systolic blood pressure [17–20]. Elevated aortic stiffness increases the hemodynamic load on the left ventricle and thereby represents one of the pre-dominant pathogenetic mechanisms leading to the development of heart failure. In the EMPA-REG OUTCOME study empagliflozin was given on top of a concomitant cardioprotective therapy. The observed reduced heart failure hospitalization and reduced cardiovascular death rate was possibly caused by a reduction in central systolic blood pressure [8]. In our clinical study arterial stiffness was reduced independent of changes in metabolic conditions but dependent on systolic blood pressure [9]. Therefore our results strengthen the role of empagliflozin as predominantly vasoprotective agent. Interestingly, clinical data revealed that the dapagliflozin mediated improvement of arterial stiffness, endothelial function and renal resistive index was independent of changes in blood pressure, suggesting a direct beneficial effect on the vasculature, possibly mediated by oxidative stress reduction [21]. Furthermore, clinical data also showed a reduction in arterial stiffness after therapy with canagliflozin [22]. Tofogliflozin has also been found to ameliorate arterial stiffness, which was associated with an improvement of liver function [23].

It is a matter of current discussion whether the reduction in mortality seen under therapy with empagliflozin is attributed to glycemic independent effects rather than glycemic control. Indeed, administration of a SGLT2 inhibitor on top of standard glucose lowering therapy modestly reduced (~ 0.4%) glycated hemoglobin plasma levels and was associated with small decreases in body weight, plasma insulin and blood pressure [8, 24]. However, it is unlikely that the small changes in these parameters can explain the large beneficial actions of SGLT2 inhibitors [25]. In vivo preclinical studies have shown decreased oxidative stress, reduced inflammatory cytokines, lowered ionic dyshomeostasis and decreased vascular and mitochondrial dysfunction after SGLT2 inhibitor administration [25–31]. Other animal studies revealed further glycemic independent effects such as the maintenance of cardiac cell viability and ATP content following hypoxia/reoxygenation in cardiomyocytes and endothelial cells [25]. In the rat model empagliflozin causes direct pleiotropic effects on the myocardium by improving diastolic stiffness and hence diastolic function [32], but it is unclear whether these effects are also present in humans, since SGLT2 is not expressed in human cardiac tissue [30]. However, there is also evidence that glycemic control by empagliflozin directly decreases macro- and micro-vascular stiffness [33]. In the mouse model hyperglycemia suppressed the anti-fibrotic factor “reversion inducing cysteine rich protein with Kazal motifs” (RECK) in the kidney, which causes an increase of renal periarterial and interstitial fibrosis [33]. This leads to an increase in renal vascular stiffness followed by an increase of aortic stiffness. Empagliflozin has been shown to ameliorate kidney injury in type 2 diabetic female mice by promoting glycosuria, and possibly by reducing systemic and renal artery stiffness, and reversing RECK suppression [33].

Our study suggests that hsCRP might be a determinant of the empagliflozin induced improvement of central pulse pressure and reflected wave amplitude. It has been previously shown in rat models that therapy with empagliflozin reduces inflammatory processes in the diabetic kidney via suppression of the advanced glycation end product receptor axis [26, 34]. In a high-fat-diet-induced obese mouse model empagliflozin reduced plasma TNF alpha levels and attenuated obesity-related chronic inflammation [35]. We now identified a relation between reduction in hsCRP and reduction of arterial stiffness in patients with early stage of type 2 diabetes mellitus. Interestingly, therapy with dapagliflozin partially reversed the formation of atherosclerosis via anti-inflammatory pathways in mice [36] and there is clinical evidence that 16 weeks of therapy with dapagliflozin reduced urine 8-hydroxy-2′-deoxyguanosine, a biomarker of oxidative stress [37]. Further clinical studies are needed to evaluate the impact of SGLT2 inhibitors on diabetes induced inflammatory processes. Especially the measurement of empagliflozin associated changes in oxidative stress parameters would we very interesting to better understand the influence of the drug on vascular function and stiffness.

The increase in copeptin levels and hematocrit measured after empagliflozin therapy compared to baseline mirrors volume depletion due to osmotic diuresis caused by glucosuria and natriuresis. It has been previously shown that SGLT2 inhibitors have a modest osmotic diuretic and natriuretic effect, which can reduce extracellular volume, blood pressure and body weight [38]. In the current analysis we did not observe a signal that volume depletion account for the improvement in arterial stiffness following empagliflozin treatment. However, a mediation analysis of the EMPA-REG OUTCOME trial identified hematocrit to be the variable with the largest impact on the hazard ratio for cardiovascular death [10]. As many participants in the EMPA-REG OUTCOME trial likely have unrecognized left ventricular dysfunction it was concluded that a key contributor to the reduction in cardiovascular death with empagliflozin was probably the change in renal sodium and glucose handling with resultant reduction in cardiac preload and ventricular stress [10]. Afterload reductions may have occurred through blood pressure and arterial stiffness lowering, thereby improving sub endocardial blood flow and reducing the risk of cardiac decompensation [10, 30]. The patients included in our clinical trial were in the early state of diabetes without evident end-organ damage. This might explain why we did not see an impact of volume status in our analysis. However, we still found an increase of hematocrit and copeptin in our study cohort. Previous studies identified complementary increased erythropoiesis as other potential mechanism to the hemodynamic changes reflected by an increase in hematocrit [39]. Additionally it has been shown that blood viscosity and shear stress in the aortic arteries increased after 3 month empagliflozin therapy in type 2 diabetic patients [40]. However, the extent to which this mechanism contributes to the cardiovascular benefits observed with empagliflozin is unclear [10]. Additionally it has to be mentioned that the volume parameters hematocrit and copeptin have known background variation. Further research based on biomarkers with less background variation and larger study cohorts is needed to evaluate the effect of volume status on the empagliflozin induced reduction of arterial stiffness.

In our study population there was a small, but significant increase in LDL- and HDL-cholesterol, which has also been previously described in the EMPA-REG-OUTCOME study [8]. This effect can be pathophysiologically explained, since reduced insulin levels caused by SGLT2 inhibition are known to trigger lipolysis by switching energy metabolism from carbohydrate to lipid utilization [38]. It has been hypothesized that this switch from carbohydrate to lipid utilization among others, such as reduced ketone clearance and stimulation of glucagon secretion, might explain the elevated risk of diabetic ketoacidosis under therapy with SGLT2 inhibitors. Diabetic ketoacidosis has been reported with the three available SGLT2 inhibitors [41]. However results from randomized controlled trials show that it is a rare event in patients with type 2 diabetes mellitus [41]. Interestingly, it was shown in animal studies, that empagliflozin reduces intestinal cholesterol absorption, which in turn promotes LDL- and macrophage-derived cholesterol fecal excretion [42]. We demonstrated in our study population that changes in LDL-levels under therapy with empagliflozin are not related to the changes in arterial stiffness.

It has been recently shown in a post hoc analysis of the EMPA-REG OUTCOME trial that changes in uric acid mediated 24.6% of the effect of empagliflozin versus placebo on the reduction in risk of cardiovascular death [10]. We therefore integrated uric acid in our analysis and found a significant decrease after 6 weeks therapy with empagliflozin compared to placebo. Nevertheless, we could not show any relation between changes in uric acid and changes in arterial stiffness.

### Limitations

One major selection bias limits the generalization of the study: only patients with normal kidney function were included and most patients with cardiovascular disease or advanced diabetes were excluded. Therefore the investigated relations only apply to patients in the early stage of diabetes without relevant end-organ damage.

The markers for volume status (copeptin and hematocrit) as well as sympathetic activation (heart rate) are only crude biomarkers with a lot of background variation, especially in a small study population like this. Therefore, based on the results of this study, it cannot be concluded that volume status and sympathetic activation are not important mediators in the effects of SGLT-2 inhibitors.

Of course, though prespecified, such an analysis of potential outcome determinants can only be the first step in the identification of factors explaining the vasoprotective potential of empagliflozin. We used placebo corrected changes in our analysis, since the differences between empagliflozin and placebo induced changes observed in the same patient entered our multiple regression analysis. The correlations between markers of vascular stiffness and hsCRP were statistically significant, but very weak and therefore clinical significance is unsure. Nevertheless, the signal that inflammation might influence empagliflozin induced reduction of arterial stiffness was present in two different statistical methods (multiple regression analysis and correlation) and might therefore be an interesting target for future investigations. Further clinical trials with detailed parameters of volume status, inflammation and sympathetic activation are needed to better understand the precise mechanism of empagliflozin-induced vasoprotection.

## Conclusion

Besides age and sex, change in systolic 24-h ambulatory blood pressure and change in hsCRP were determinants of the empagliflozin induced improvement of vascular parameters of arterial stiffness, whereas parameters of change in glucose metabolism, lipid metabolism, uric acid, sympathetic activation as assessed by heart rate and volume status had no significant influence. Our analysis found a signal that empagliflozin may exert, at least to some extent, its beneficial vascular effects via anti-inflammatory mechanisms.

## Abbreviations

ACE-inhibitor: angiotensin converting enzyme inhibitor
BP: blood pressure
cAIx: central augmentation index
cAIx@75: cAIx normalized to a heart rate of 75 beats per minute
CHF: congestive heart failure
eGFR: estimated glomerular filtration rate
HDL: high density lipid
hsCRP: high sensitive C-reactive protein
HR: heart rate
LDL: low density lipid
SD: standard deviation
SGLT2-inhibitor: sodium-glucose cotransporter 2 inhibitor
